# Modulation of the Volatile Profile of Cardamom (*Elettaria cardamomum*) Essential Oil by Non‐Thermal Instant Controlled Pressure Drop (DIC) Technology: A Novel Approach in Food Processing

**DOI:** 10.1002/fsn3.71395

**Published:** 2026-01-09

**Authors:** Giselle Dení Teresa‐Martínez, Patricia Rodriguez‐Castillo, Maritza Alonzo‐Macías, Carmen Téllez‐Pérez, Anaberta Cardador‐Martínez

**Affiliations:** ^1^ Tecnologico de Monterrey Escuela de Ingeniería y Ciencias San Pablo Mexico; ^2^ Intensification of Transfer Phenomena on Industrial Eco‐Processes, Laboratory of Engineering Science for Environment, University of La Rochelle, LaSIE—UMR‐CNRS 7356 La Rochelle France

**Keywords:** antioxidants, cardamom, essential oil, GC–MS, hydrodistillation, instant controlled pressure drop

## Abstract

*Elettaria cardamomum* (cardamom) is an Asian‐origin fragrant seed highly valued for its sensory attributes, including its delicate scent and distinctive flavor. Essential oils (EOs) constitute the primary extracts obtained from cardamom, and their market demand has increased considerably in recent years, largely due to their recognized antioxidant properties. To support this growing demand, innovative technologies such as instant controlled pressure drop (DIC) have been explored. DIC increases cell porosity, promoting solvent accessibility and improving extraction efficiency while preserving heat‐sensitive constituents, an advantage when coupled with conventional methods like hydrodistillation, which remains the standard method for EO production. GC–MS analysis identified 14 volatile components in the extracted oils. Limonene was the predominant compound, reaching 40.54% under DIC 7 (122°C, 19 s), followed by eucalyptol, which reached 24.31% under DIC 8 (122°C, 41 s). A combination of DIC processing parameters influenced the composition of key constituents. For compounds such as 3‐carene, eucalyptol, linalyl valerate, ρ‐menth‐1‐en‐4‐ol, and α‐citral, both processing time and temperature contributed to their variation. In contrast, other compounds, such as ρ‐mentha‐1,4‐dien‐7‐ol, α‐terpineol, β‐selinene, and geraniol, were driven primarily by a single factor. Overall, this study shows that DIC pretreatment can potentially modulate the volatile profile of cardamom essential oil and highlights how specific DIC conditions influence the abundance of individual key constituents.

## Graphical abstract

This work highlighted the potential of instant controlled pressure drop technology as a possible modulator of specific compounds within complex plant extracts such as essential oil. Both time and temperature had important effects on the yield of specific compounds, underscoring the importance of tailoring them to maximize metabolites of interest. Among some of the major components like eucalyptol, this pretreatment meant an increase in the obtention yield, highlighting this technology mechanistic advantages.

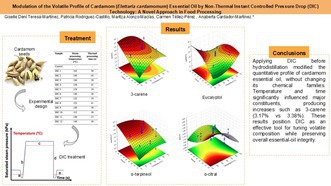

## 1 Introduction

Cardamom, often known as the “Queen of Spices,” is a fragrant asian‐origin spice. This plant is highly esteemed for its distinctive aroma and its versatile use in both cooking and traditional medicine (Ikeda et al. 1962; Ivanović et al. 2021; Nigam et al. 1965). Cardamom is cultivated in multiple nations, including India, Guatemala, Mexico, and Tanzania, with Guatemala leading global production and India ranking a close second (K Ashokkumar et al. 2020; Sengottuvelu 2011).

Cardamom has long been employed in culinary and medicinal contexts, it has been widely used for centuries in Indian Ayurveda, where it is recognized for its therapeutic relevance (Al‐Zuhair et al. 1996; Zachariah 2002). Assyrian physicians and chemists used a diverse array of medicinal herbs, including cardamom, for therapeutic purposes (Masoumi‐Ardakani et al. 2016; Sahal et al. 2024). In Greece and Rome, in an time when spices were regarded as markers of affluence and high standing, cardamom held historical significance (Masoumi‐Ardakani et al. 2016). Even today, Cardamom remains a versatile ingredient, widely incorporated into cosmetic formulations and used across both the culinary and pharmaceutical sectors (Sobhy et al. 2025; Teymuri‐Yeghaneh et al. 2025).

Cardamom essential oils (EOs) are primary extracts obtained from seeds; they are highly valued mainly due to their antibacterial activity (Souissi et al. 2020), antifungal activity (Noumi et al. 2018), antidiabetic activity (Aghasi et al. 2018), and antioxidant activities. In recent years, the global demand for natural extracts and essential oils has increased significantly, driven by consumer interest in plant‐based food additives, cosmetics, and functional ingredients (Machado et al. 2022; Ramsey et al. 2020). Essential oils have extensive applications across various sectors, including cosmetics, food and beverage, and aromatherapy (Irshad et al. 2019; Sobhy et al. 2025; Teymuri‐Yeghaneh et al. 2025). Essential oil demand was 226.8 kt in 2018, and it is projected to reach 399 kt by 2035. Moreover, the value of the essential oils market is predicted as USD 49.07 billion by 2033 (Irshad et al. 2019; StraitsResearch 2025). Along with the previously stated properties, antioxidant, antispasmodic, antimicrobial, and carminative properties of essential oils are intrinsically linked to their unique chemical profiles (Espina et al. 2011; Fornari et al. 2012; Irshad et al. 2019).

In this contex, essential oils (EOs) are secondary plant metabolites that are lipophilic and volatile (Baser and Buchbauer 2009). According to the International Organization for Standardization (ISO), an EO is defined as a “product obtained from a natural raw material of plant origin, by steam distillation, mechanical processes from the epicarp of citrus fruits, or dry distillation, after separation of the aqueous phase—if any—by physical processes” (ISO 2013). An EO is essential because it contains the essence of the different fragrances and properties of the plant from which they are extracted (de Sousa et al. 2023). EOs have been integral to human civilization for over three millennia, playing central roles in traditional medicine, ceremonial rituals, and the crafting of perfumes (Irshad et al. 2019).

EOs are mainly composed of monoterpenes; which are considered secondary metabolites since they are not essential for plant viability; however, these compounds mediate important interactions concerning plants and the environment (Bakkali et al. 2008; Baptista‐Silva et al. 2020; Baser and Buchbauer 2009). Research has shown that monoterpenes exhibit a diverse spectrum of pharmacological activities, including antifungal, antioxidant, antibacterial, anticancer, antiviral, local anesthetic, antihistaminic, anti‐inflammatory, and antispasmodic effects. Monoterpenes also act as growth regulators, tumor inhibitors, inhibitors of oxidative phosphorylation, and insect repellents (Maurya et al. 2021).

Traditionally, hydrodistillation has been used to extract essential oils. In this technique, the vapor pressures of the substances reach equilibrium with the surrounding air pressure at the boiling temperature. The vapor carrying the target compounds rises and enters a slender tube, which is externally cooled, typically with cold water or an antifreeze solution, o induce condensation. As a result, the steam condenses and is collected in a container. Due to the lower density of the essential oil compared to water, it rises while the water sinks (Bousbia 2011; Chemat and Boutekedjiret 2015). This process offers advantages such as low equipment costs and simplicity. However, it also has drawbacks, including high energy consumption and a lengthy processing period, which can lead to undesirable chemical alterations in the essential oil. To overcome these limitations, novel extraction‐enhancing technologies have been explored. Instant Controlled Pressure Drop technology, or DIC for its acronym in French, has been developed. In terms of energy usage, a study by Allaf et al. (2013) found that, when comparing hydrodistillation (HD) DIC for extracting orange peels, DIC technology significantly reduces energy consumption once a certain extraction efficiency threshold is reached.

Conceived initially to prevent structural collapse and improve the texture of bioproducts materials during drying operations, the Instant Controlled Pressure Drop (DIC) method rapidly evolved to serve diverse sectors, such as food processing, pharmaceuticals, cosmetics, and EOs extraction, enabling applications like decontamination and phytochemical extraction (Allaf et al. 2016). DIC is a thermomechanical method that involves subjecting the sample to high‐pressure saturated steam (ranging from 0.1 to 1.0 MPa) for a brief duration of time, typically a few seconds, followed by an immediate pressure drop to near vacuum conditions (around 30 mbar) (Téllez‐Pérez et al. 2019). The sudden drop in pressure towards a vacuum induces the auto‐vaporization of water, causes the matrix to swell, and potentially weakens the cell walls. This, in turn, facilitates enhanced solvent diffusion within the solid material and improves mass transfer, thereby significantly accelerating the extraction kinetics of DIC‐treated substances (Mounir et al. 2014; Téllez‐Pérez et al. 2019). Given these characteristics, there is a possibility that combining DIC with hydro distillation (HD) technology could lead to increased not only essential oil extraction efficiency but also modify its chemical composition.

As far as we are aware, no prior investigation has been conducted into the utilization of DIC technology as a pretreatment method for cardamom seeds prior hydrodistillation and its effect on essential oil composition. Therefore, this comparative study examines the impact on the essential oil profile when using the conventional hydrodistillation method alone versus coupling DIC with hydrodistillation.

## 2 Materials and Methods

### 2.1 Materials

The cardamom seeds, with an initial moisture content of 5.14% d.b, were procured from Finca Argovia, located in Tapachula, Chiapas, México. The solvents used in this study were HPLC‐grade and obtained from Sigma‐Aldrich (St. Louis, MO, USA).

### 2.2 Methods

#### 2.2.1 Moisture Content

The seed moisture level was determined using a laboratory air dryer (Binder FD 23) by a gravimetric method (Pomeranz and Meloan 1994). Two grams of seeds were dried in a controlled environment at 105°C until constant weight was achieved. Moisture was determined post DIC treatment.

#### 2.2.2 Experimental Design and Statistical Analysis for DIC Pretreatment

Response surface methods were applied using a central composite design, resulting in 12 trials, including 4 center points (refer to Table 1). This study focused primarily on the essential oil (EO) profile. The experimental variables analyzed were the saturated steam temperature (°C) and the duration of thermal treatment (seconds).

**TABLE 1:** Experimental design of the DIC pretreatment parameters.

Sample	Steam processing temperature (°C)	Thermal processing time (s)	Moisture content (%, d.b.)
Control	—	—	5.14
DIC 1	165	30	11.27
DIC 2	140	45	10.90
DIC 3	140	30	11.16
DIC 4	158	41	11.33
DIC 5	158	19	11.04
DIC 6	140	30	10.59
DIC 7	122	19	10.80
DIC 8	122	41	10.50
DIC 9	140	30	10.83
DIC 10	115	30	10.83
DIC 11	140	15	10.74
DIC 12	140	30	10.64

To assess the impact of experimental variables in the DIC design, the statistical analysis used both Pareto charts and response surface methodology. In the Pareto chart, a vertical line was used to identify effects that were statistically significant at the 95% confidence level.

All the experiments were conducted in triplicate and the data are presented as mean values of triplicate determinations. Statistical analysis of the data was performed by analysis of variance (ANOVA); the level of statistical significance was set at *p* < 0.05. Only response‐surface models with *R* ^2^ > 0.5 were retained for interpretation; all fitted models exhibited a lack‐of‐fit value greater than 0.05.

All analyses were performed using Statistica Software 2017 (TIBCO Software Inc., Palo Alto, CA, USA).

#### 2.2.3 DIC Pretreatment

**FIGURE 1:**
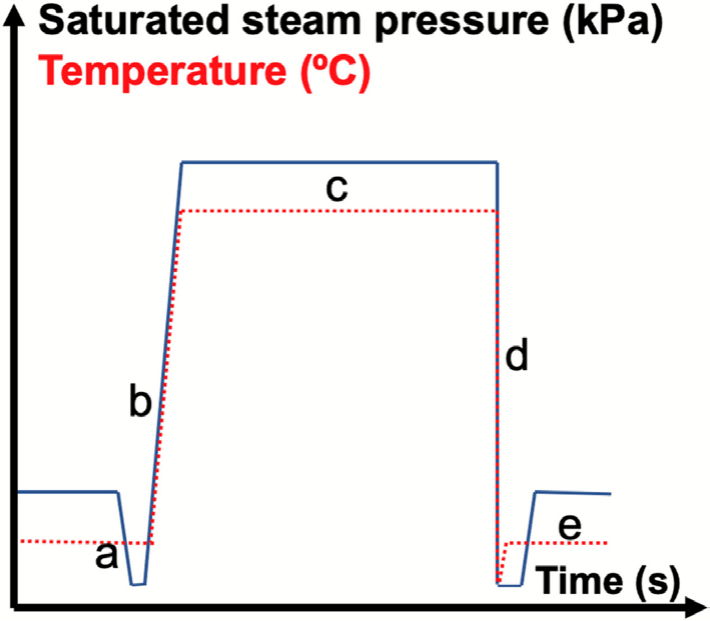
General diagram of a DIC cycle, where: (a) is the establishment of an initial vacuum in the processing vessel; (b) is the injection of saturated dry steam at the selected pressure; (c) is the maintenance of treatment pressure during the selected time; (d) is the instant controlled pressure drop towards a vacuum; and (e) is the release to atmospheric pressure. *Y* axis is the saturated steam pressure (kPa) and *X* axis is processing time (s).

The DIC pretreatment of cardamom seeds involved four steps. Figure 1 shows a schematic representation of a DIC processing cycle. Initially, 100 g of seeds were placed inside the DIC reactor, which was then subjected to a vacuum of 30 mbar (Figure 1a). Subsequently, as shown in Figure 1b,c, saturated steam was injected into the reactor until reaching the desired saturated steam temperature ranging from 115°C to 165°C (equivalent to 0.17 to 0.7 MPa), and the temperature was held briefly (ranging from 15 to 45 s). Following this, the samples experienced an instant controlled pressure drop (∆*P*/∆*t* > 0.5 MPa·s^−1^) towards a vacuum (30 mbar) (Figure 1d). This pressure drop‐induced auto‐vaporization of the water, resulting in matrix swelling. Lastly, the pressure was released to return to atmospheric pressure (Figure 1e), and the cardamom seeds were recovered. The DIC equipment utilized in this study was the DIC MP model (ABCAR‐DIC Process, La Rochelle, France). Following DIC treatment, the cardamom seeds were stored at −80°C until further analysis.

#### 2.2.4 Essential Oil Extraction via Hydrodistillation

This study mixed 50 g of ground cardamom (35 mesh size particles) with 600 mL of distilled water using a Clevenger‐type apparatus. The resulting combination was distilled for 6 h. The essential oil obtained was then dried with anhydrous sodium sulfate and stored at −80°C, protected from light, until further examination (Chemat and Boutekedjiret 2015). The essential oil yield was determined by measuring the mass of oil obtained per 100 g of seed, based on the seed's dry weight, as previously reported (Teresa‐Martínez et al. 2022).

#### 2.2.5 GC–MS Analysis

Gas chromatographic analyses of volatile compounds were conducted using an Agilent 7890A (Santa Clara, California) equipped with a flame ionization detector. Separation of the compounds was achieved using an HP‐FFAP capillary column with dimensions of 50 m length, 0.20 mm internal diameter, and 0.30 mm thickness (Agilent). The oven program consisted of an initial temperature of 55°C for 6.5 min, followed by a ramping rate of 10°C/min until reaching 165°C, then a further increase of 30°C/min until reaching 220°C, where it was held for 5 min. The injector temperature was set at 220°C, while the detector temperature was maintained at 240°C. For each analysis, an automatic injection of 0.5 mL of the sample was performed, utilizing helium as the carrier gas at a flow rate of 1.0 mL/min. Identification was carried out by comparing their mass spectra with a NIST database. No internal standards were included for this determination.

## 3 Results and Discussion

### 3.1 Essential Oil Analysis by Gas Chromatography–Mass Spectrometry (GC–MS)

To assess how DIC pretreatment alters the chemical profile of essential oils, GC–MS analysis was conducted to identify the main groups of constituents in each experimental condition. The components discovered in the different treatments are presented in Table 2. A total of 14 volatile components were identified across all samples. The chemical compounds that were found in the highest quantities were monoterpenes, monoterpenoids, sesquiterpenes, and acetate esters. The findings presented in this study resembled the outcomes published by Alam et al. (2021) and Tarfaoui et al. (2022), where both studies identified nearly identical components in the essential oil of cardamom.

**TABLE 2:** Essential oil compounds identified by GC–MS in cardamom essential oil.

Compound ID	CAS number	Classification
3‐carene	13,466‐78‐9	Monoterpene hydrocarbon
ρ‐Mentha‐1,4‐dien‐7‐ol	22,539‐72‐6	Monoterpene oxygenated
β‐myrcene	123‐35‐3	Monoterpene
Eucalyptol	470‐82‐6	Monoterpenoid
Linalyl acetate	115‐95‐7	Monoterpenoid
Linalyl valerate	10,471‐96‐2	Monoterpenoid
ρ‐Menth‐1‐en‐4‐ol	562‐74‐3	Menthane monoterpenoid
Limonene	138‐86‐3	Monoterpene hydrocarbon
α‐terpineol	98‐55‐5	Monoterpene alcohol
β‐selinene	17,066‐67‐0	Sesquiterpene
α‐citral	5392‐40‐5	Monoterpene oxygenated
Nerol acetate	141‐12‐8	Acetate ester
Geraniol	106‐24‐1	Monoterpenoid
Nerolidol	40,716‐66‐3	Sesquiterpenoid

**FIGURE 2:**
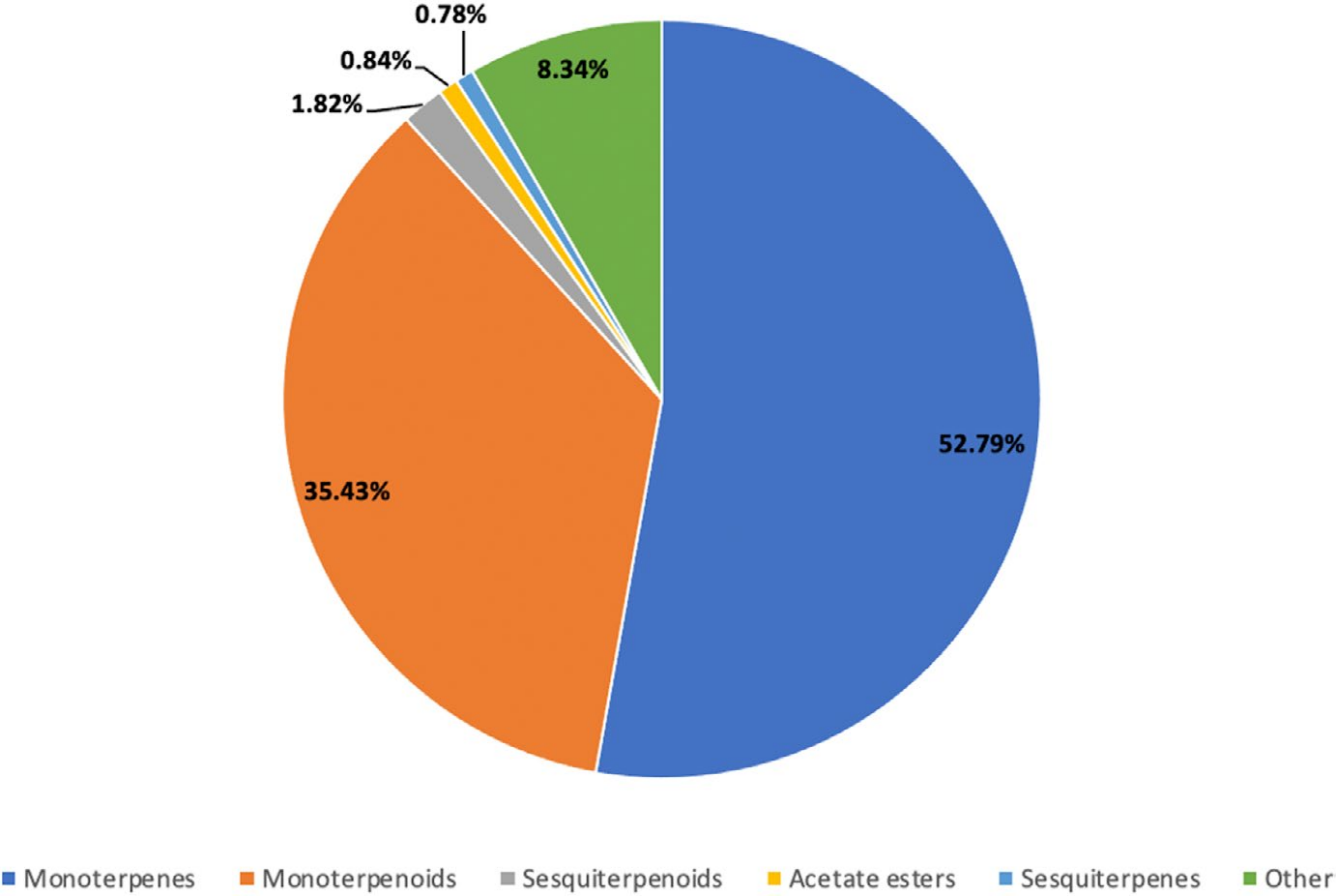
Composition of cardamom essential oil by chemical family.

The results of this study indicated that the use of DIC pretreatment did not produce substantial changes in the chemical composition of the essential oils (EOs), as assessed by the families of compounds found in the samples (Table 2). The observed uniformity in the chemical profile may be attributable to the slight influence of the rapid cycles of the DIC pretreatment, which effectively maintains the stability of these compounds (Table 3). Based on the composition percentages shown in Figure 2, the predominant constituents of the essential oil were monoterpenes, accounting for approximately 52.8% of the total composition. The subsequent components observed were monoterpenoids, accounting for 35.43% of the total composition, followed by sesquiterpenoids at 1.81%, acetate esters at 0.835%, and sesquiterpenes at 0.78%. By contrast, Noumi et al. (2018) and Kaliyaperumal Ashokkumar et al. (2021) reported oxygenated monoterpenes (63% and 81%, respectively) as the main components of cardamom essential oil. According to Stolle et al. (2009), high temperatures and long hydrodistillation times can cause oxidation of monoterpenes. In this study, although the essential oil was obtained by hydrodistillation, the DIC pretreatment could have improved extraction efficiency, possibly preventing the conversion of monoterpenes to oxygenated compounds by reducing oxidation.

**TABLE 3:** Table of area % of the compounds identified in essential oils from *E. cardamomum* identified by gas chromatography–mass spectrometry.

			Sample												
Compound	Peak #	RI	CON‐TROL	DIC 1	DIC 2	DIC 3	DIC 4	DIC 5	DIC 6	DIC 7	DIC 8	DIC 9	DIC 10	DIC 11	DIC 12
3‐Carene	1	1180	3.17	2.78	3.38	3.33	3.15	3.05	3.09	2.76	3.19	2.95	3.02	2.87	3.19
ρ‐Mentha‐1,4‐dien‐7‐ol	2	1274	3.87	3.60	3.97	4.08	3.93	3.99	4.11	3.98	4.04	3.84	4.10	3.90	3.98
β‐myrcene	3	1180	2.45	2.18	2.45	2.62	2.57	2.67	2.60	2.53	2.62	2.53	2.63	2.53	2.63
Eucalyptol	4	1190	24.13	24.17	23.04	23.38	23.29	23.51	23.84	22.84	24.31	22.72	23.80	22.18	23.78
Linalyl acetate	5	1569	7.31	6.77	7.14	7.09	6.96	6.19	6.98	6.86	7.27	6.86	7.02	6.94	6.84
Linalyl valerate	6	1570	2.68	2.61	2.19	2.43	2.21	2.52	2.60	2.74	2.06	2.45	2.16	2.44	2.32
ρ‐Menth‐1‐en‐4‐ol	7	1650	2.55	2.46	2.45	2.53	2.42	2.54	2.38	2.40	2.53	2.51	2.51	2.60	2.59
Limonene	8	1229	37.65	37.85	38.39	38.43	39.16	39.31	38.88	40.54	38.58	39.41	38.03	39.39	38.18
α‐terpineol	9	1732	0.74	1.73	2.71	2.20	2.47	1.49	1.85	0.97	2.46	2.16	2.42	1.68	2.21
β‐selinene	10	1781	0.78	0.70	0.64	0.77	0.67	0.91	0.78	0.95	0.78	0.89	0.68	0.86	0.88
α‐citral	11	1240	1.13	0.99	1.18	1.18	1.19	1.22	1.19	1.27	1.13	1.26	1.25	1.28	1.24
Nerol acetate	12	1728	0.87	0.78	0.84	0.87	0.80	0.90	0.85	0.83	0.87	0.55	0.84	0.99	0.90
Geraniol	13	1760	1.53	1.30	1.54	1.48	1.48	1.46	1.39	1.36	1.59	1.41	1.50	1.58	1.45
Nerolidol	14	2055	2.00	1.67	1.97	1.80	1.88	2.04	1.77	1.90	1.67	1.80	1.87	2.06	1.20

*Note:* Results were calculated based on the chromatogram's relative peak area (percent area); this results are given as the average of triplicate determinations. RI (retention index) was determined relative to the retention time of a series of n‐alkanes (C5–C34) on a HP‐FFAP column.

**FIGURE 3:**
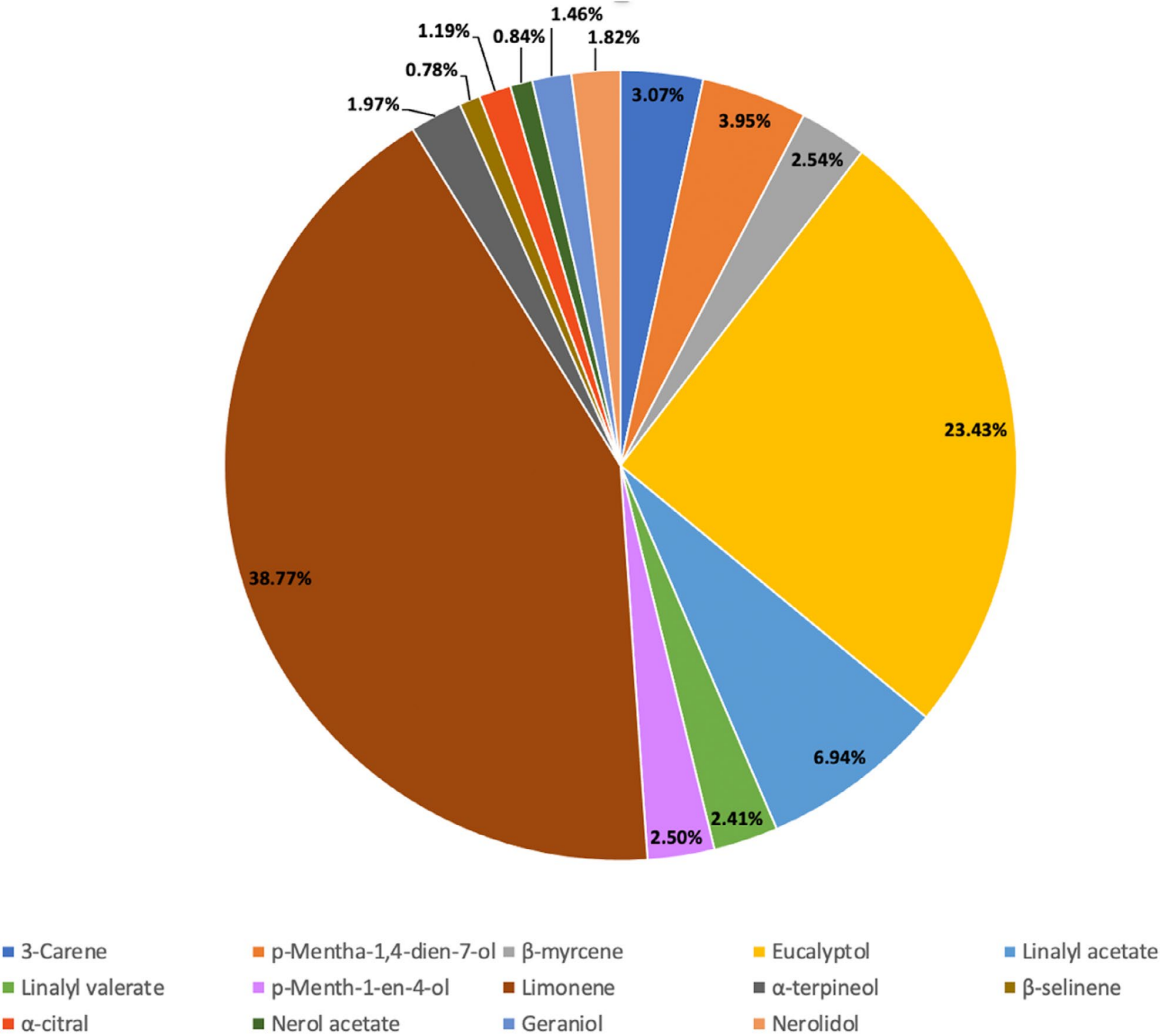
Composition of cardamom essential oil by components (average across DIC‐treated samples).

**FIGURE 4:**
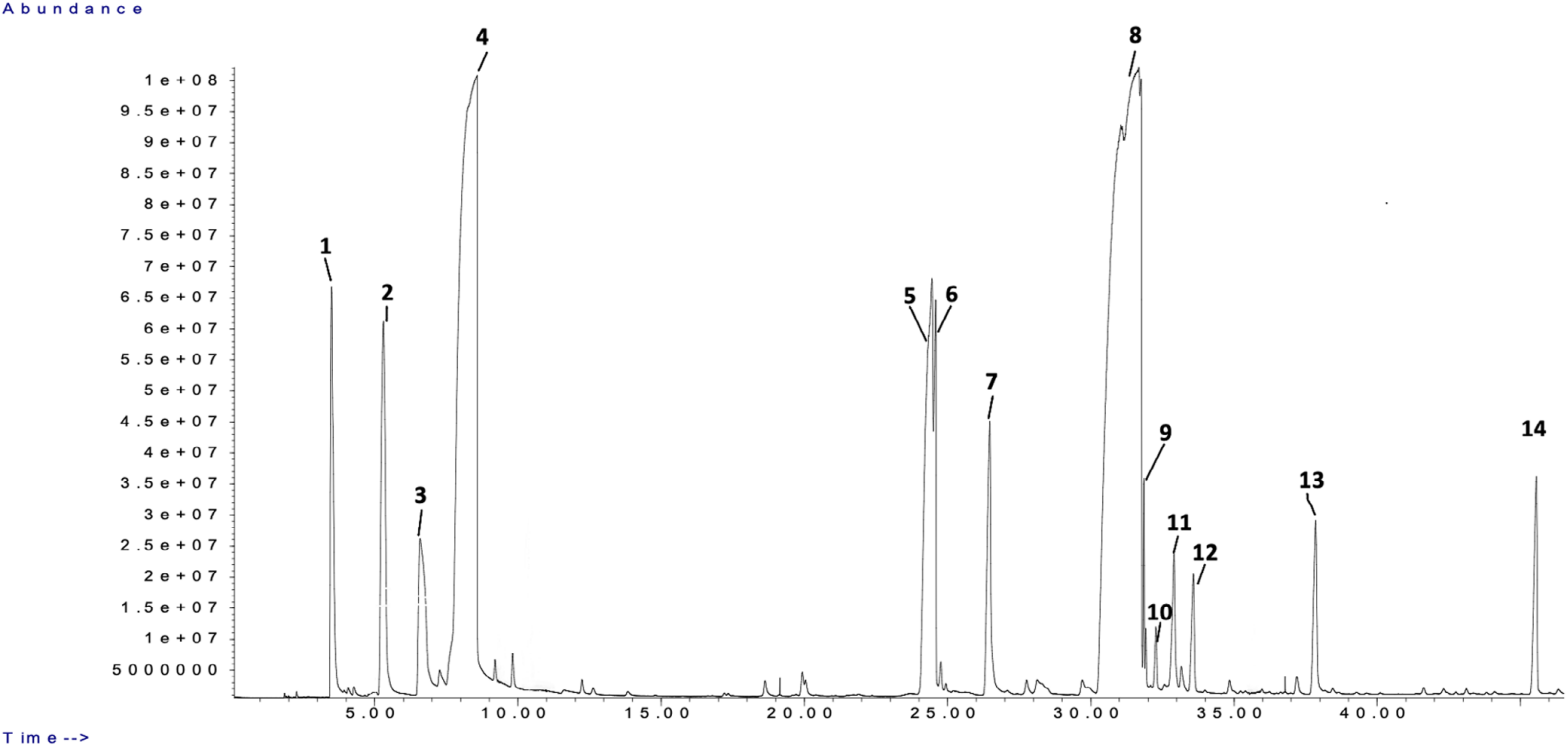
Chromatogram of *Elettaria cardamomum* essential oil obtained under DIC 9 conditions. Numbers (1–14) correspond to the individual volatile compounds identified and listed in Table 3. Peaks 5 and 6 represent two co‐eluting constituents resolved by mass‐spectral matching.

Limonene was the predominant constituent among the monoterpenes in all samples, with percentages ranging from 37.65% to 40.54% for DIC 7 (Figure 3). This finding contrasts with previous studies by Molaveisi et al. (2020) and Abdullah et al. (2022), which examined essential oils obtained through hydrodistillation without coupling with DIC. In those studies, the main compound identified was α‐terpinyl acetate, with percentages of 33.07% and 42.6%, respectively. It is worth noting that α‐terpinyl acetate is an oxygenated monoterpene, while limonene is a non‐oxygenated compound with less complexity. Figure 4 shows the representative GC chromatographic profile of the essential oil. It is worth noting that this approach only provides semi‐quantitative results rather than absolute quantification of the components of cardamom essential oil.

Furthermore, these observations imply that combining DIC with hydrodistillation may increase the occurrence of certain constituents in the essential oil, leading to a distinct, predominant chemical compound: limonene. However, it is important to note that the composition of essential oils (EOs) may vary by region of origin and the specific plant from which they are derived. The identification of monoterpenes in the essential oils (EOs) obtained by distillation with instant controlled pressure drop (DIC) suggests the possibility of enhanced therapeutic properties compared to EOs obtained by alternative methods.

It is essential to note that some monoterpenes possess substantial antioxidant activity (Abdullah et al. 2022; Noumi et al. 2018), which may explain the observed antioxidant activity of cardamom essential oil. In prior studies, this team found that the AOX capacity of cardamom seeds treated with DIC varied from 62.28% to 68.18% (Teresa‐Martínez et al. 2022). This discovery contrasts with the results reported by Ramadan et al. (2022), who achieved an AOX range of 26% to 32%. The observed variation can be attributed to the DIC and its positive influence on AOX. Despite the significant importance of these advancements, additional studies are required to determine the precise mechanism by which DIC increases the antioxidant activity of cardamom essential oils. Additionally, a thorough examination of the possible synergistic effects of DIC in conjunction with other chemicals or botanical extracts could provide a deeper understanding of its capacity to enhance antioxidant qualities.

In GC–MS profiling, cardamom essential oil is dominated by non‐phenolic monoterpenes such as limonene (≈38%) and eucalyptol (≈24%), which lack classic phenolic rings, but act as antioxidants primarily via a termination‐enhancing mechanism, stabilizing peroxyl radicals (Amorati et al. 2013; Amorati and Valgimigli 2018). By contrast, phenolic terpenes (e.g., carvacrol, thymol) operate via a chain‐breaking mechanism by hydrogen‐atom donation to peroxyl radicals (Amorati et al. 2013).

Cravero et al. (2024) demonstrated that at 60°C an oregano–laurel blend (rich in phenolic terpenes) matched BHT's inhibition of sunflower‐oil oxidation over 28 days, confirming the predominance of chain‐breaking action under moderate heat. Conversely, at 150°C López et al. (2023) found that oregano and hop EOs (rich in non‐phenolic terpenes such as terpinen‐4‐ol and β‐myrcene) still delivered up to an 81% PV reduction versus control after 8 h, underscoring the resilience of termination‐enhancing protection under frying conditions.

Together, these data suggest that combining a phenolic‐rich EO (for chain‐breaking at lower temperatures) with cardamom's non‐phenolic EO (for termination‐enhancing at high temperatures) could afford synergistic, broad‐spectrum antioxidant protection in thermal food applications.

In thermal treatments such as DIC, the normal boiling point (BP) of an essential‐oil constituent is the primary driver of its volatility: compounds with lower BPs vaporize faster at higher temperatures, whereas higher‐BP compounds remain in the liquid phase. In this case, the GC–MS profile of cardamom oil shows that the major monoterpenes, limonene (BP 176°C) and eucalyptol (BP 176°C–177°C), span this volatility range. Thus, during DIC pretreatment, lower‐BP constituents will flash‐evaporate to a greater extent, leaving a residual oil enriched in higher‐BP compounds. This behavior mirrors Olmedo et al. (2014). Findings for oregano oil, where short‐path molecular distillation at 25°C under vacuum produced distillate fractions enriched in low‐BP terpenes and residue fractions enriched in higher‐BP components such as terpinen‐4‐ol and carvacrol. Such selective volatilization likely underlies the differentiated antioxidant outcomes we observe: preferential loss of low‐BP, more volatile antioxidants can reduce radical‐scavenging capacity at moderate temperatures, whereas concentration of higher‐BP compounds preserves antioxidant efficacy under high‐temperature oxidation.

**FIGURE 5:**
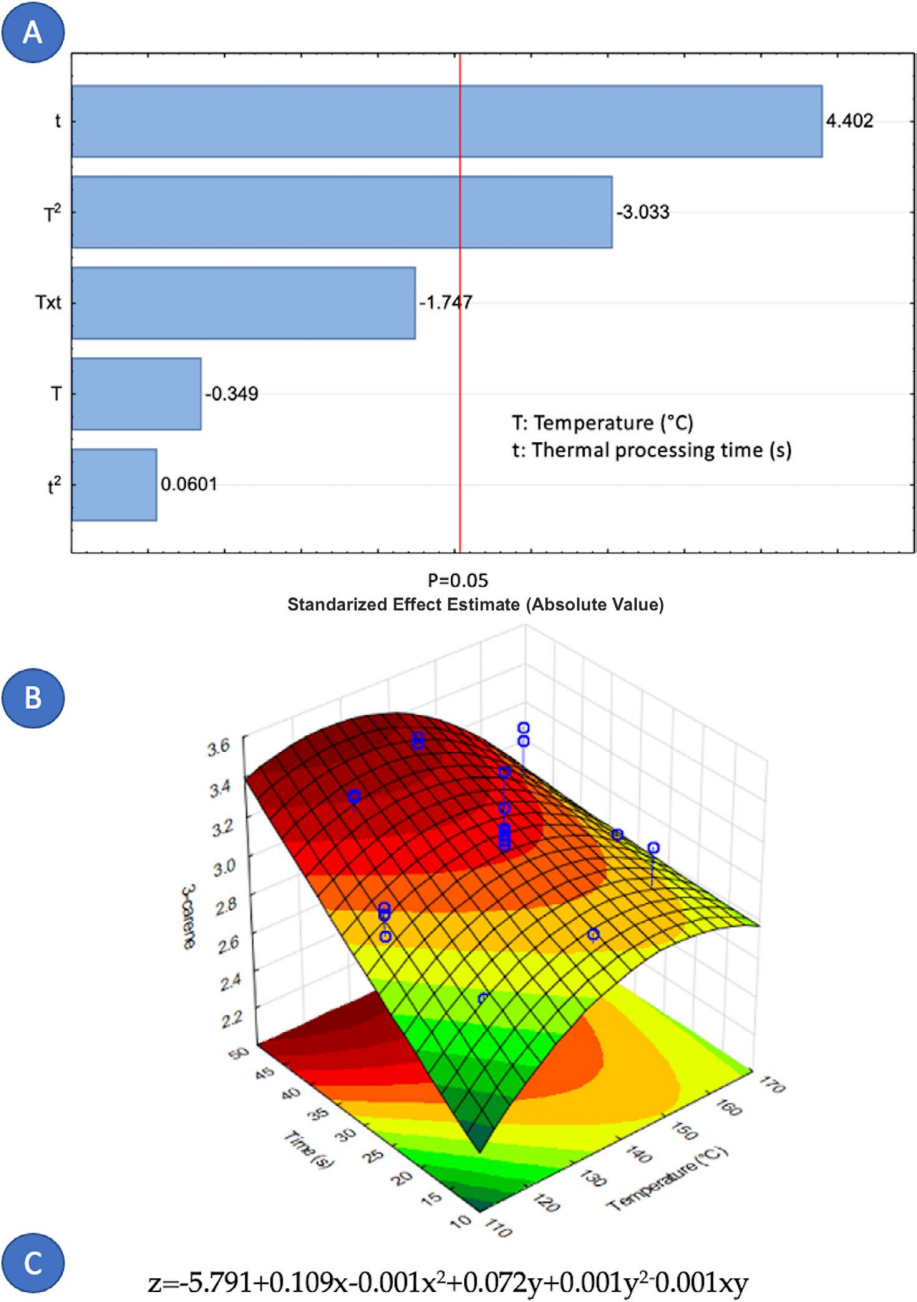
Effect of steam processing temperature “*T*” (°C) and thermal processing time “*t*” on yield of 3‐carene. (A) Pareto chart (B) Surface response (C) equation where: *z* = 3‐carene, *x* = temperature, and *y* = time.

**FIGURE 6:**
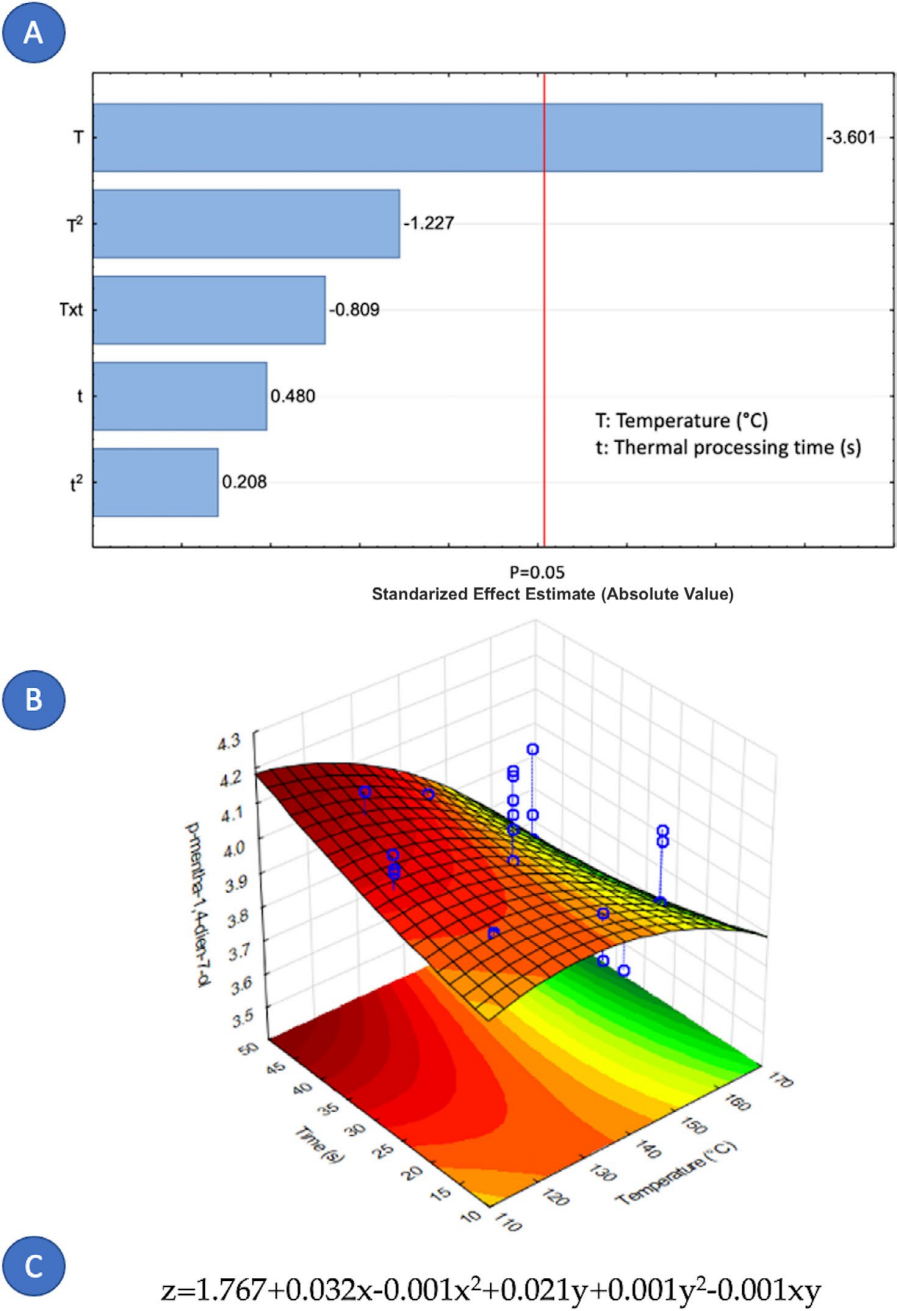
Effect of steam processing temperature “*T*” (°C) and thermal processing time “*t*” on yield of p‐Mentha‐1,4‐dien‐7‐ol. (A) Pareto chart (B) Surface response (C) equation where: *z* = p‐Mentha‐1,4‐dien‐7‐ol, *x* = temperature, and *y* = time.

**FIGURE 7:**
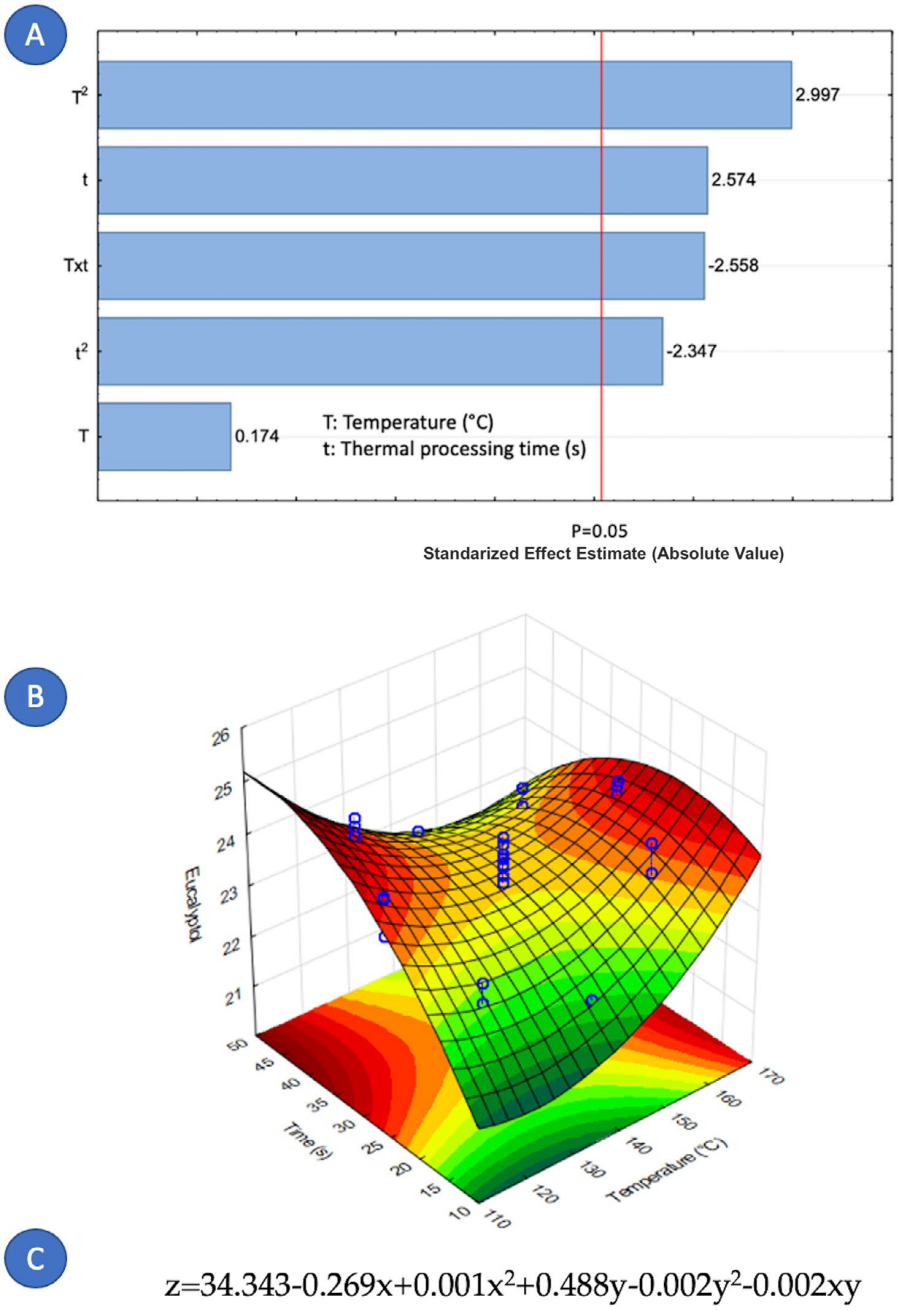
Effect of steam processing temperature “*T*” (°C) and thermal processing time “*t*” on yield of Eucalyptol. (A) Pareto chart (B) Surface response (C) equation where: *z* = Eucalyptol, *x* = temperature, and *y* = time.

**FIGURE 8:**
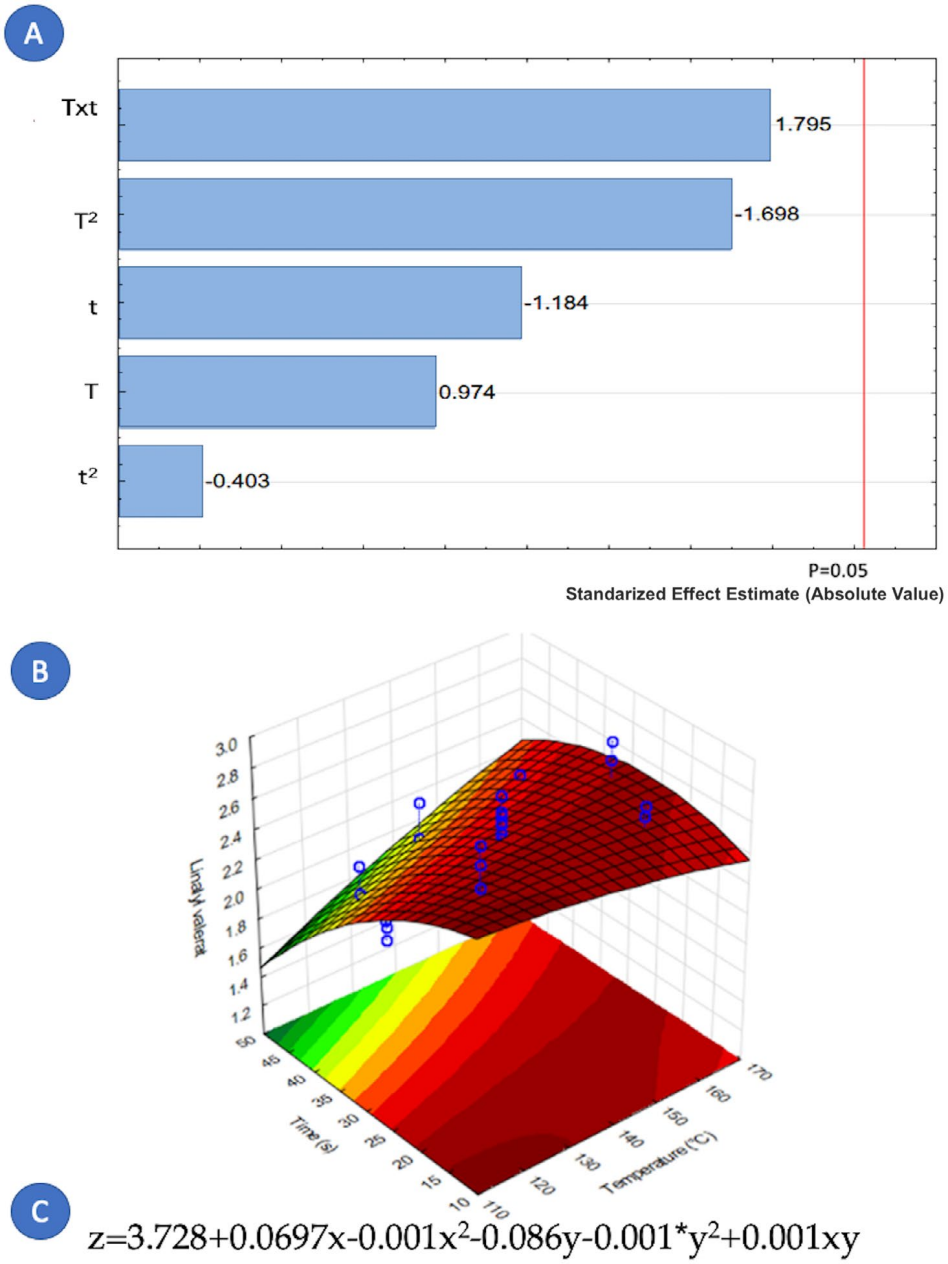
Effect of steam processing temperature “*T*” (°C) and thermal processing time “*t*” on yield of Linalyl valerate. (A) Pareto chart (B) Surface response (C) equation where: *z* = Linalyl valerate, *x* = temperature, and *y* = time.

**FIGURE 9:**
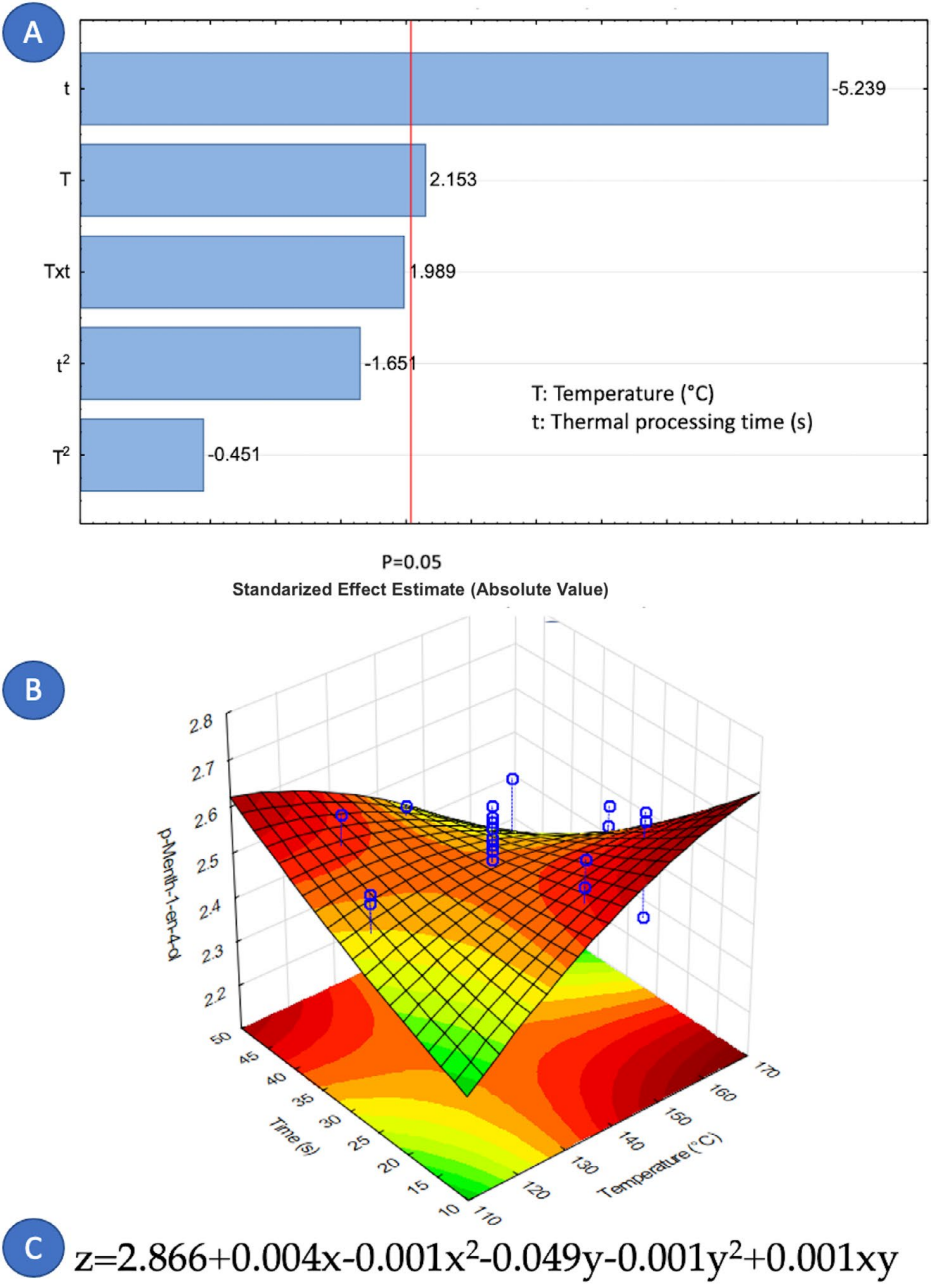
Effect of steam processing temperature “*T*” (°C) and thermal processing time “*t*” on yield of p‐Menth‐1‐en‐4‐ol. (A) Pareto chart (B) Surface response (C) equation where: *z* = *p*‐Menth‐1‐en‐4‐ol, *x* = temperature and *y* = time.

**FIGURE 10:**
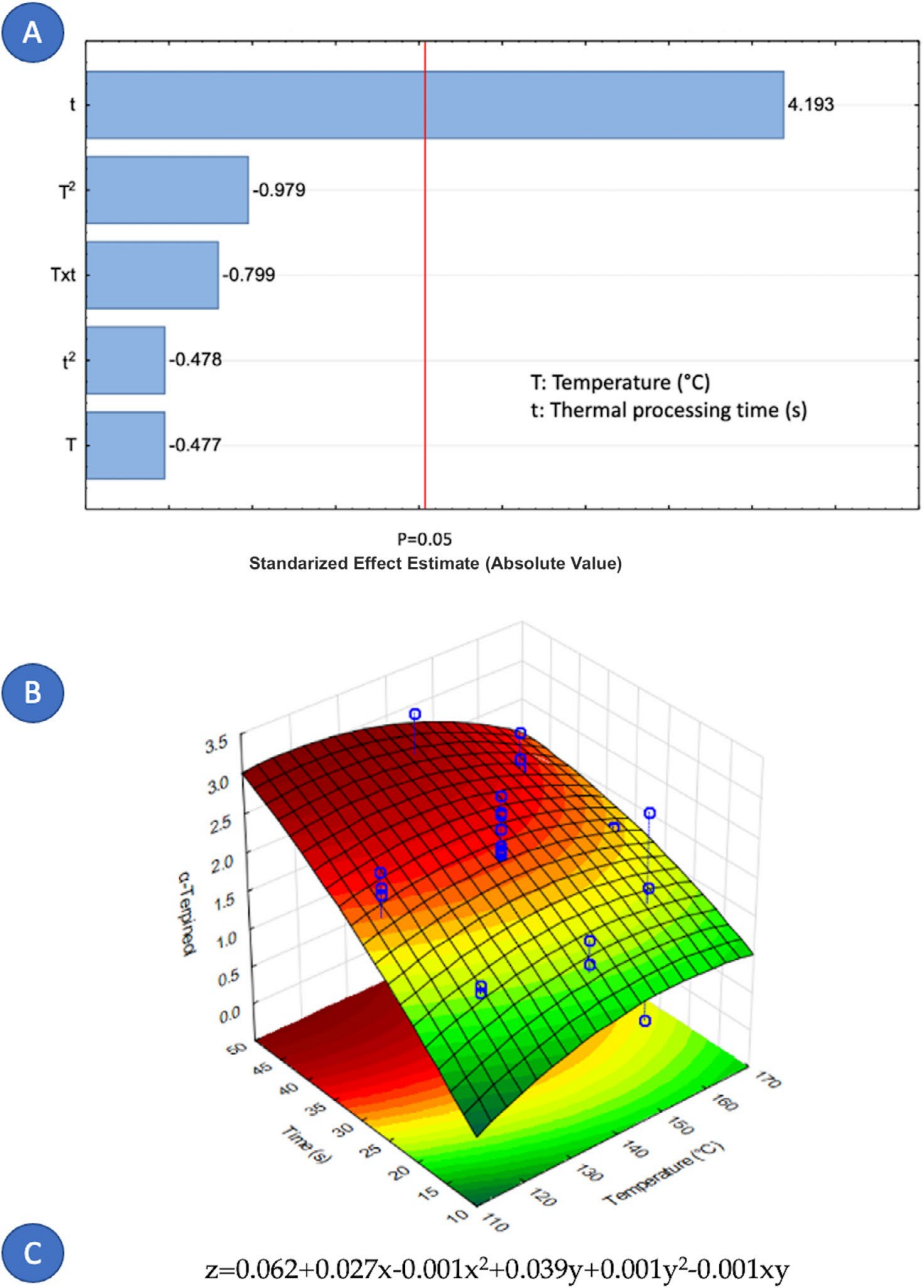
Effect of steam processing temperature “*T*” (°C) and thermal processing time “*t*” on yield of α‐terpineol. (A) Pareto chart (B) Surface response (C) equation where: *z* = α‐terpineol, *x* = temperature and *y* = time.

**FIGURE 11:**
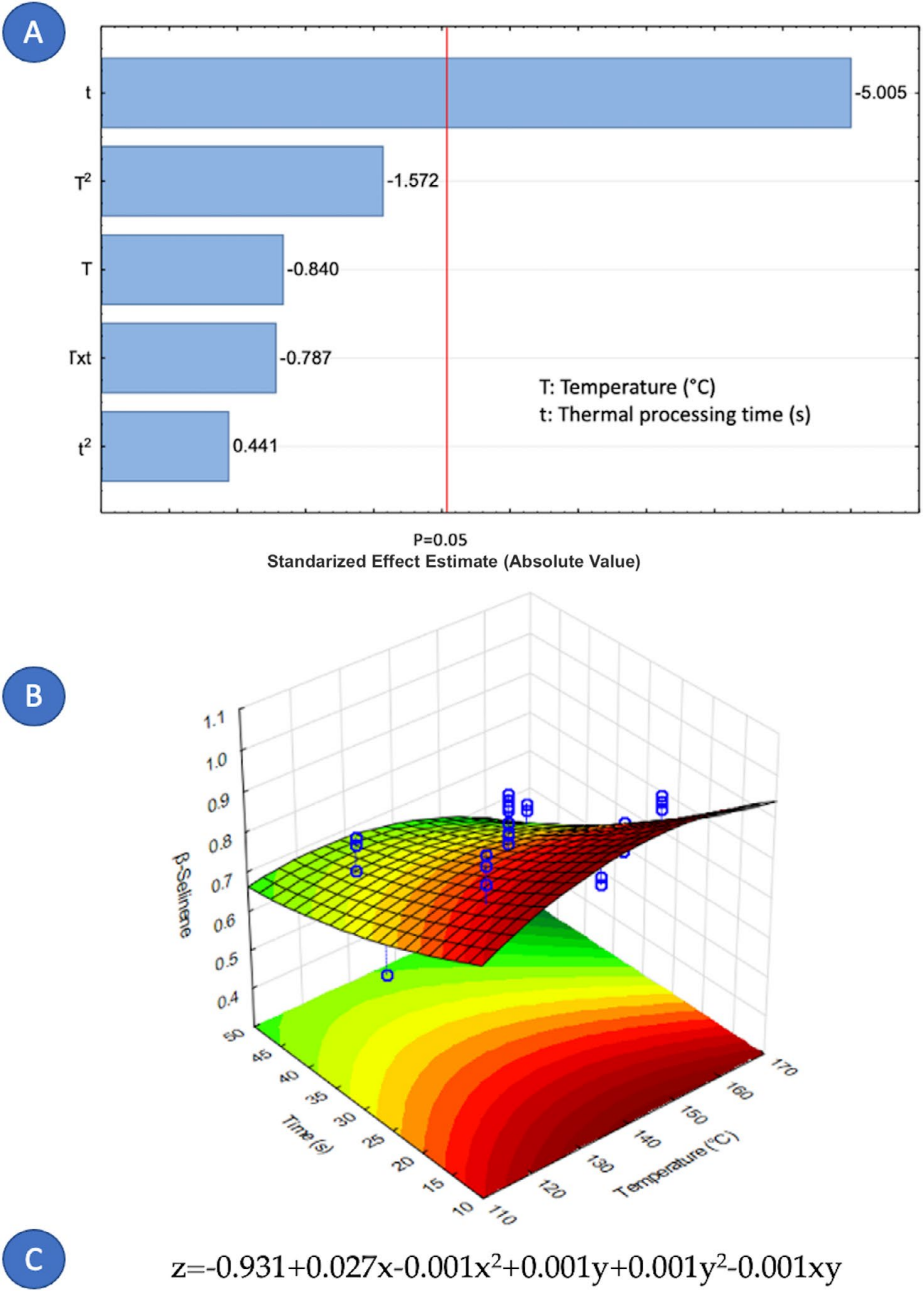
Effect of steam processing temperature “*T*” (°C) and thermal processing time “*t*” on yield of β‐selinene. (A) Pareto chart (B) Surface response (C) equation where: *z* = β‐selinene, *x* = temperature, and *y* = time.

**FIGURE 12:**
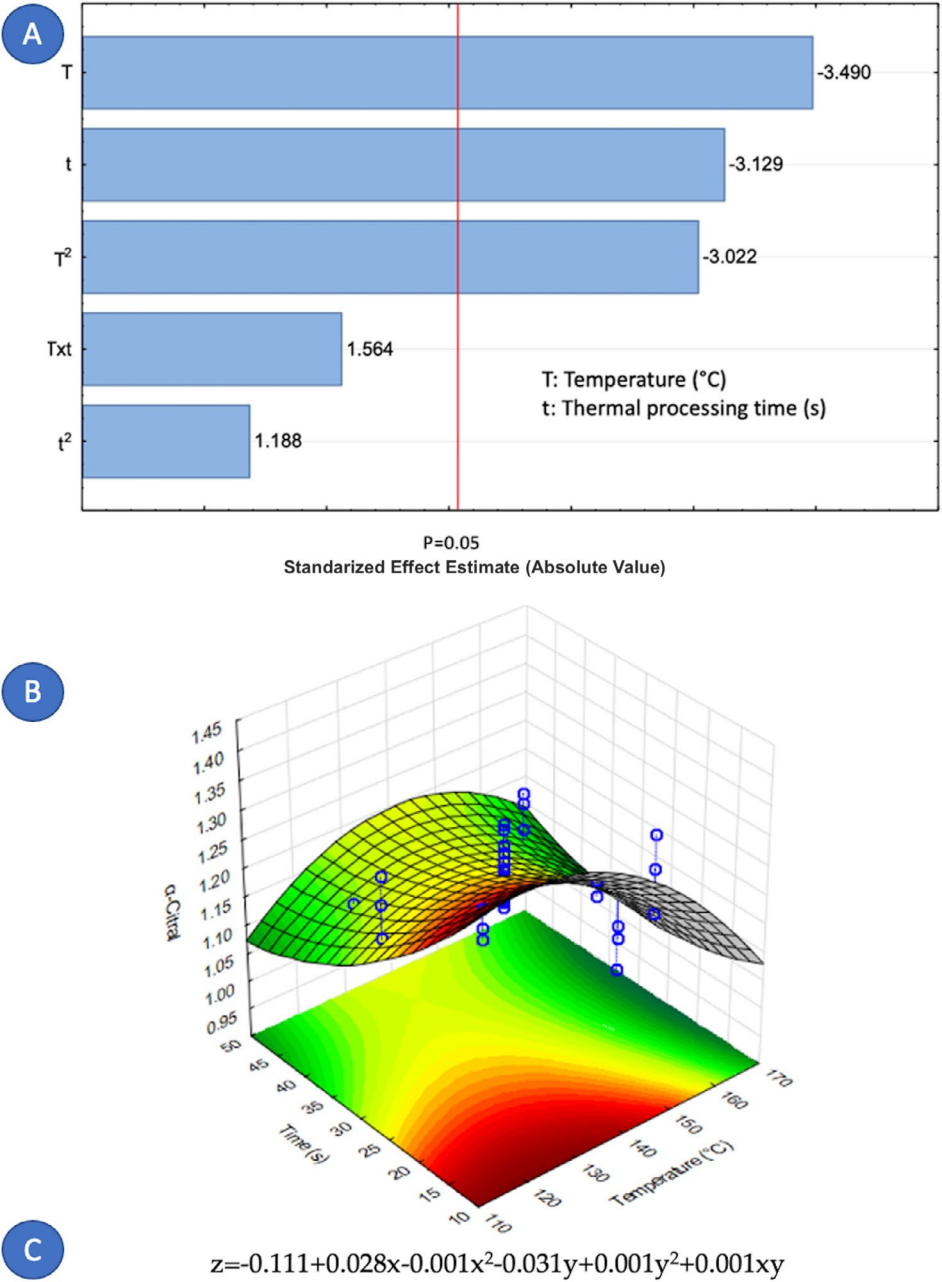
Effect of steam processing temperature “*T*” (°C) and thermal processing time “*t*” on yield of α‐citral. (A) Pareto chart (B) Surface response (C) equation where: *z* = α‐citral, *x* = temperature and *y* = time.

**FIGURE 13:**
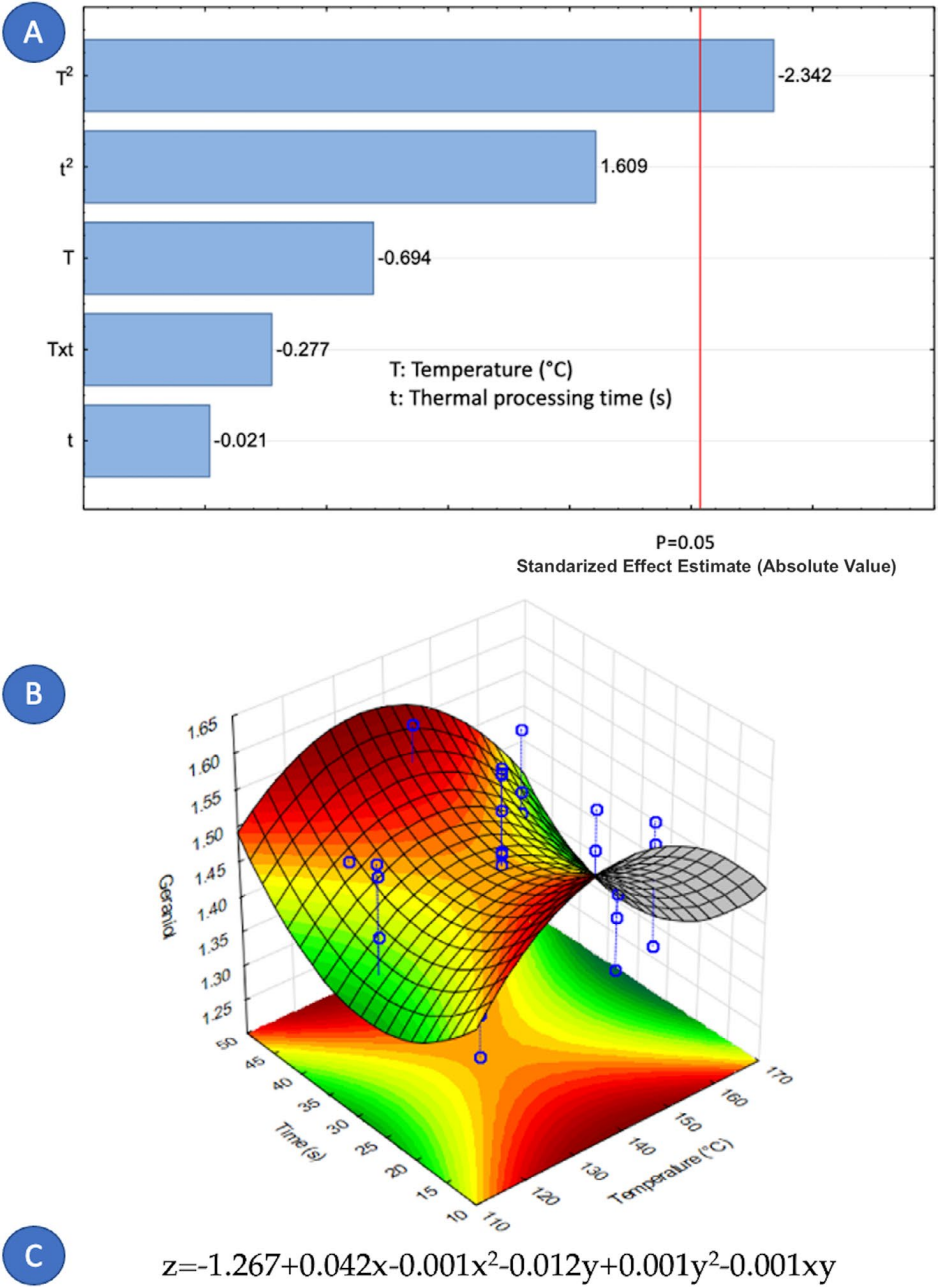
Effect of steam processing temperature “*T*” (°C) and thermal processing time “*t*” on yield of Geraniol. (A) Pareto chart (B) Surface response (C) equation where: *z* = Geraniol, *x* = temperature and *y* = time.

Concerning the factors that influence the composition of cardamom essential oil, it is notable that only nine out of fourteen components were affected by the variables under study. Figures 5, 6, 7, 8, 9, 10, 11, 12, 13 illustrate how the steam processing temperature “*T*” and thermal processing time “*t*” influence the yield of each individual component. The Pareto charts (Figures 5, 6, 7, 8, 9, 10, 11, 12, 13) highlight which factors exert statistically significant effects, revealing that both temperature and time impact five specific components. Meanwhile, the corresponding response surface plots (Figures 5, 6, 7, 8, 9, 10, 11, 12, 13) provide a mathematical representation of the relationships among temperature, time, and yield. In Figures 5, 6, 7, 8, 9, 10, 11, 12, 13, the equations are given explicitly, with *z* representing the yield of the respective compound, *x* denoting temperature (°C), and *y* representing time (s). The Pareto charts reveal that for most components, a combination of factors influences their composition. This is the case for components such as 3‐carene (Figure 5A), eucalyptol (Figure 7A), ρ‐Menth‐1‐en‐4‐ol (Figure 9A), and α‐citral (Figures 8 and 12A).

Conversely, there are components where a single factor holds the principal influence, such as ρ‐Mentha‐1,4‐dien‐7‐ol (Figure 6A), α‐terpineol (Figure 10A), β‐selinene (Figure 11A), and geraniol (Figure 13A). Additionally, it is important to note that processing time is the predominant component of the EO. However, in most cases, this relationship is negative, indicating that an increase in processing time leads to a decrease in the corresponding component. This should be considered when designing the treatment, ensuring a balance between the desired enhancement of specific components and their potential reduction.

Overall, longer exposure combined with low to moderate temperatures meant an increase in the yields of compounds like 3‐carene, ρ‐Mentha‐1,4‐dien‐7‐ol, eucalyptol, ρ‐Menth‐1‐en‐4‐ol, α‐terpineol, and geraniol, which meant a rise from 1.9 to more than 100% in the most successful cases such as α‐terpineol.

The previously included mechanistic explanations are provided as a theoretical framework. No antioxidant assays were performed in this study; these descriptions should be interpreted solely as background supporting the discussion of compositional variation.

Thus, this interpretation relies on the quantitative GC–MS results. Although DIC did not alter the qualitative chemical families present in cardamom EO, several constituents showed clear shifts in relative abundance. For example, limonene increased from 37.65% in the control to 40.54% under DIC 7. Similarly, 3‐carene rose from 3.17% (control) to 3.38% under DIC 2, while eucalyptol rose from 24.13% to 24.31% under DIC 8. Esters such as linalyl valerate increased from 2.68% to 2.74% under DIC 7. Other compounds exhibited selective responses; for instance, ρ‐menth‐1‐en‐4‐ol decreased from 2.55% (control) to 2.38% under DIC 6. These observations show that DIC can potentially act as a selective modulator of compounds within the cardamom EO profile, altering the abundance of specific constituents while preserving the overall family distribution.

## 4 Conclusions

Historically, the process of obtaining essential oil from cardamom seeds has been achieved by hydrodistillation (HD). Nevertheless, this approach is accompanied by several drawbacks, including increased energy consumption and the potential thermal degradation of volatile organic compounds during prolonged extraction times. To examine these concerns, the present work has investigated the effects of integrating DIC (Instant Controlled Pressure Drop) technology with HD on the efficacy of essential oil extraction and its associated biological characteristics.

Fourteen volatile components were identified in the extracted cardamom essential oil using gas chromatography–mass spectrometry (GC–MS). The applied treatment influenced the composition of each major component. For 3‐carene (*t* and *T* ^2^), eucalyptol (*T* ^2^, *t*, Txt and *t* ^2^), linalyl valerate (*t* and *T*), ρ‐Menth‐1‐en‐4‐ol (*t* and *T*), and α‐citral (*T*, *t* and *T* ^2^), both studied independent variables, the treatment temperature (*T*) and the treatment time (*t*) were significant. On the contrary, for ρ‐Mentha‐1,4‐dien‐7‐ol (*T*), α‐terpineol (*t*), β‐selinene (*t*), and geraniol (*T* ^2^), only one independent variable exerts an influence. Furthermore, the element that holds the most significance among the various components of the EO was the processing time. The present study demonstrates that DIC pretreatment influences the quantitative composition of cardamom essential oil without altering its qualitative chemical families. Several constituents exhibited measurable changes under specific DIC conditions, supporting the role of DIC as a selective modulator of volatile composition. For example, limonene increased from 37.65% in the control to 40.54% under DIC 7, representing an approximate 7.7% relative increase. Likewise, 3‐carene increased from 3.17% to 3.38% under DIC 2 (a 6.6% relative increase), while eucalyptol rose from 24.13% to 24.31% under DIC 8. Esters such as linalyl valerate increased from 2.68% to 2.74% under DIC 7, whereas ρ‐menth‐1‐en‐4‐ol decreased from 2.55% to 2.38% under DIC 6. These shifts show that DIC can modify the proportions of specific volatile constituents while preserving the overall structural categories in the EO profile.

This provides a thought‐provoking foundation for treatment design, emphasizing the importance of selecting optimized DIC treatment conditions to achieve an ideal balance between the processing duration and the required improvement in specific components, while ensuring the integrity of their total amount and quality. Maintaining this intricate equilibrium could facilitate achieving the intended result in DIC treatment while simultaneously mitigating any potential diminishment in these important constituents and their overall quality.

## Author Contributions

**Giselle Dení Teresa‐Martínez:** writing – original draft, investigation, methodology, formal analysis. **Patricia Rodriguez‐Castillo:** methodology, writing – original draft, data curation. **Maritza Alonzo‐Macías:** statistical analysis, software, resources, visualization, supervision, writing – review and editing. **Carmen Téllez‐Pérez:** conceptualization, visualization, supervision, writing – review and editing, formal analysis, data curation. **Anaberta Cardador‐Martínez:** conceptualization, writing – review and editing, supervision, funding acquisition.

## Conflicts of Interest

The authors declare no conflicts of interest.

## Supporting information


**Data S1:** fsn371395‐sup‐0001‐Supinfo1.docx.

## Data Availability

The data that support the findings of this study are available from the corresponding author upon reasonable request.
