# Supplementary material for: Modulation of the Volatile Profile of Cardamom (Elettaria cardamomum) Essential Oil by Non‐Thermal Instant Controlled Pressure Drop (DIC) Technology: A Novel Approach in Food Processing
Source: Food Sci Nutr. 2026 Jan 9;14(1):e71395. doi: 10.1002/fsn3.71395 (PMC12784164; doi:10.1002/fsn3.71395)
Supplement: Supplementary file 1 — Data S1: fsn371395‐sup‐0001‐Supinfo1.docx. [file FSN3-14-e71395-s001.docx]

Grapchical abstract


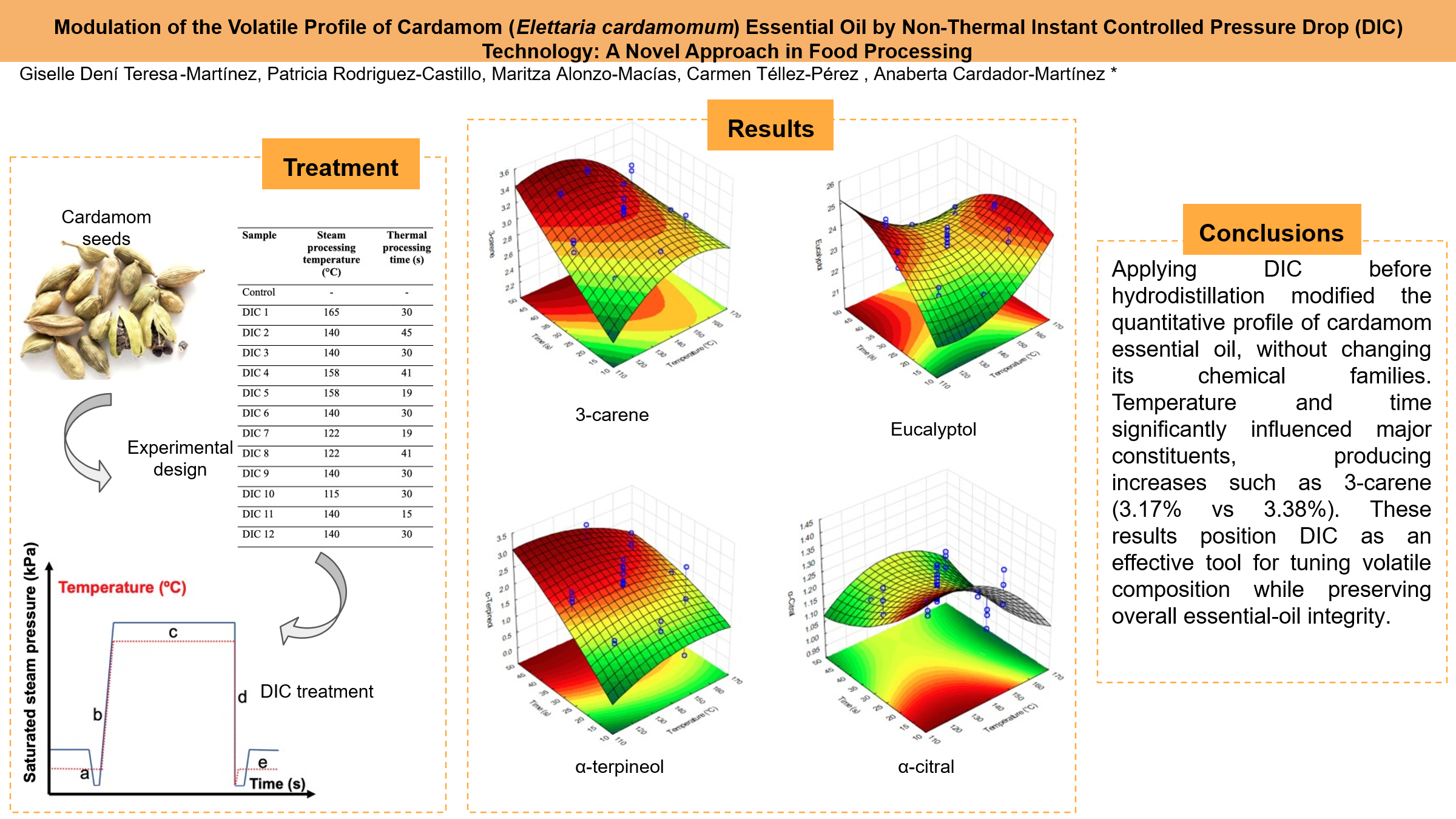


Main findings.

- This work highlighted the potential of instant controlled pressure drop technology as a possible modulator of specific compounds within complex plant extracts such as essential oil.
- Both time and temperature had important effects on the yield of specific compounds, underscoring the importance of tailoring them to maximize metabolites of interest.
- Among some of the major components like eucalyptol, this pretreatment meant an increase in the obtention yield, highlighting this technology mechanistic advantages.
